# Nightmares as predictors of suicide: an extension study including war veterans

**DOI:** 10.1038/srep44756

**Published:** 2017-03-15

**Authors:** Nils Sandman, Katja Valli, Erkki Kronholm, Erkki Vartiainen, Tiina Laatikainen, Tiina Paunio

**Affiliations:** 1 Center for Cognitive Neuroscience, Turku Brain and Mind Center, Department of Psychology and Speech-Language Pathology, University of Turku, Turku, Finland; 2 Genomics and Biomarkers Unit, National Institute for Health and Welfare, Helsinki, Finland; 3 Department of Cognitive Neuroscience and Philosophy, School of Bioscience, University of Skövde, Skövde, Sweden; 4 Department of Health, National Institute for Health and Welfare, Turku, Finland; 5 Department of Health, National Institute for Health and Welfare, Helsinki, Finland; 6 Institute of Public Health and Clinical Nutrition, University of Eastern Finland, Kuopio, Finland; 7 Hospital District of North Karelia, Joensuu, Finland; 8 Department of Psychiatry, University of Helsinki and Helsinki University Central Hospital, Helsinki, Finland

## Abstract

Nightmares are intensive dreams with negative emotional tone. Frequent nightmares can pose a serious clinical problem and in 2001, Tanskanen *et al*. found that nightmares increase the risk of suicide. However, the dataset used by these authors included war veterans in whom nightmare frequency - and possibly also suicide risk - is elevated. Therefore, re-examination of the association between nightmares and suicide in these data is warranted. We investigated the relationship between nightmares and suicide both in the general population and war veterans in Finnish National FINRISK Study from the years 1972 to 2012, a dataset overlapping with the one used in the study by Tanskanen *et al*. Our data comprise 71,068 participants of whom 3139 are war veterans. Participants were followed from their survey participation until the end of 2014 or death. Suicides (N = 398) were identified from the National Causes of Death Register. Frequent nightmares increase the risk of suicide: The result of Tanskanen *et al*. holds even when war experiences are controlled for. Actually nightmares are not significantly associated with suicides among war veterans. These results support the role of nightmares as an independent risk factor for suicide instead of just being proxy for history of traumatic experiences.

Diagnostic and Statistical Manual of Mental Disorders, Fifth edition (DSM-V) defines nightmares as extremely dysphoric and well-remembered dreams that usually involve efforts to avoid threats to survival, security, or physical integrity^1^. While occasional nightmares are common and usually do not lead to long-term negative consequences, around 5% of adults suffer from frequent nightmares^2^,^3^,^4^. Frequent nightmares have been linked to other sleep problems, especially insomnia, and mental health problems, especially symptoms of depression, as well as to lowered quality of life in general^3^,^5^,^6^,^7^,^8^,^9^.

Nightmares are also a defining symptom of post-traumatic stress disorder (PTSD)^10^. PTSD is a condition that may develop after exposure to traumatic event, for example, death, violence, serious injury or sexual violence. The person developing PTSD may be subject of the traumatic event, witness it or encounter it in a professional setting. Symptoms of PTSD include repeated re-experiencing of the trauma which typically take the form of intrusive thoughts, nightmares and flashbacks, but also avoidance of trauma related stimuli, and depressive and anxiety symptoms^1^. The prevalence of PTSD is elevated among populations that have high risk of encountering traumatic events, for example war veterans, and these populations also have higher prevalence of nightmares than the general population^2^.

In 2001, Tanskanen *et al*.^11^ published a study on nightmares as predictors of suicide in Finland, where suicide rate has been high (25–45 per 100 000 person years for men) during the last four decades^12^. They used a series of population level health surveys, FINRISK, to obtain information on self-estimated nightmare frequency and linked this data to the Finnish National Causes of Death Register which contains information on suicides. They found that men who had frequent nightmares had a significantly higher risk of suicide (Hazard Ratio (HR) 1.66 [1.15–2.39]) than those who did not have nightmares. Among women, similar trend (HR 1.22 [0.57–2.60]) emerged, but it lacked statistical significance due to the small number of suicides (n = 30) by women in the dataset.

In addition to the study of Tanskanen *et al*.^11^, several other studies have investigated the association between nightmares and suicidality. These have been summarized in Table 1. The methods and study populations of these studies vary: Many studies have used clinical samples and therefore their results may not reflect the general population. However, most studies report a significant link between nightmares and either suicidal thoughts as measured by questionnaires or suicide attempts. In addition to nightmares, disturbed sleep in general and insomnia in particular increase the risk for suicidality^13^,^14^,^15^,^16^,^17^,^18^,^19^,^20^.

As nightmares are strongly associated with depression and PTSD, we agree with Tanskanen *et al*.^11^ that “The common denominator between the frequency of nightmares and an increased risk for suicide may be a history of trauma”. In our earlier study^2^ in which we utilized the same FINRISK data used by Tanskanen *et al*., we noticed a sizeable and identifiable population of veterans of the Second World War (WWII) in these data. Compared to the general population with nightmare prevalence of 3.5% among men, the war veterans had elevated nightmare frequency of 7–11%, even 30 or more years after the war had ended. We hypothesise that this elevated nightmare frequency among the veterans is related to post-traumatic nightmares caused by war experiences. There is also evidence that war veterans, especially those with PTSD, have increased risk for suicidality, although the evidence is mostly based on studies from the Vietnam era or later^21^,^22^,^23^,^24^.

The sample of the original study by Tanskanen *et al*.^11^ included veterans of the Second Word War (WWII), but they were not identified and analysed separately from the general population. Thus, the possible effect that war veterans may have had on the association between nightmares and suicide was not explored - a state of affairs we set out to remedy.

The aim of the current study was thus to perform an extension of the original study by Tanskanen *et al*.^11^. We analysed partially overlapping data from the FINRISK series of health surveys linked to the National Causes of Death Register, as in the original study, with three significant improvements:

First, we identify war veterans in the data and investigate whether war experiences are a more significant predictor of suicide than nightmare frequency by controlling for war experiences in the analyses between nightmares and suicide. We also explore this association among the veteran population separately.

Second, we study risk factors for nightmares among war veterans and compare them with the risk factors for nightmares in the general population.

Third, our sample size is 91% larger and the follow-up period 9 years longer than in the study of 2001, equalling 191% more person-years and resulting in increased statistical power.

We test the following two hypotheses: First, we predict that the association between nightmares and suicide becomes markedly less significant in the whole sample when the war veterans are excluded from the study population. Second, we expect that among war veterans nightmares are a stronger predictor of suicide than among the general population, as post-war nightmares often stem from serious mental health issues, including PTSD and major depression.

## Methods

### FINRISK survey

The Finnish National FINRISK Study is a series of health surveys conducted with representative cross-sectional random population samples of Finnish adults. The first survey was carried out in 1972 and the series has been ongoing, conducted every five years, with the latest survey completed in 2012. Every FINRISK survey included a comprehensive health questionnaire and a health examination in a local health care centre. For more information on the sampling methods for FINRISK, see Borodulin *et al*.^25^.

Each FINRISK study has been conducted in accordance with the ethical and legal guidelines of the time of survey and informed consent was obtained from all participants (verbal in 1972–1992 and written since 1997). The most recent ethical committee to give approval for FINRISK studies (2002–2012) was the ethical committee of Hospital District of Helsinki and Uusimaa and the study methods complied with the Declaration of Helsinki. Participants of FINRISK had also been asked for the permission to link the FINRISK questionnaire answers with the National Causes of Death Register, and only those who consented are included in the sample of the current study.

FINRISK data contains sensitive personal information about the participants and is not publicly available. Additional information about FINRISK data can be found on study website^26^ and inquiries about the data can be addressed to the FINRISK steering committee at the Department of Health at the Finnish National Institute for Health and Welfare.

### Sample

In the original study by Tanskanen *et al*.^11^, data from the surveys of 1972, 1977, 1982, 1987, and 1992 (N = 36 211) was used. Participants of the surveys were linked to the Causes of Death Registry of Finland with their social security number and followed from participation in the survey until the end of 1995 or death (522 150 person-years).

In the current study we utilized the same data as Tanskanen *et al*.^11^ with addition of four new surveys from 1997, 2002, 2007, and 2012. Our sample included 71 068 participants with an age range of 25–74 years (mean 45.6, SD 12.39) 51.6% of whom are women. The FINRISK data were again linked to the Causes of Death Registry and participants followed until 31 December 2014 or death (1 521 327 person-years).

The FINRISK surveys of 1972 and 1977 included a question: “Did you serve on the front during the last wars? Yes/no” and a follow-up question “Were you wounded during the war” with answer options “no”, “yes, slightly (no permanent injury)” and “yes, I am a war invalid.” The question referred to Winter War (1939–40) and Continuation War (1941–44) during WWII when Finland fought the Soviet Union.

Among the participants of FINRISK 1972 and 1977, this question identified 3139 men who had served at the front during WWII. When analysed separately, these men were followed from their participation in the survey until the end of 2014 or death (65 630 person years). There were 163 women who answered yes to “Serving at the front” question, but their numbers were too low to conduct any meaningful analyses with regards to suicide rates.

After 1977 there were no questions about war experiences in FINRISK. Finland has not been involved in armed conflicts since WWII, so the number of participants with war experiences from other conflicts in our data is minimal. However, in surveys of 1982–1987 and 1997 there are participants (5223 men and 5991 women) with birth year of 1926 or earlier. These participants were at least 18 years old in 1944 and thus some of them are war veterans, but from the data available it is impossible to identify whom exactly. In some analyses we study the whole generation of participants born before 1927 as a group with potential war experiences. The different groups of participants are presented in Table 2.

For further information about the prevalence of nightmares among general population and war veterans in FINRISK, please refer to Sandman *et al*.^2^.

### Measures

The question about nightmares in FINRISK reads as follows: “*During the past 30 days have you had nightmares?* ” with answer options “*often*”, “*sometimes*” *and* “*never.”* The participants were not given any definition of a nightmare. This question was identical in all FINRISK surveys and 69 101 participants of the total of 71 068 had answered this question.

As co-variates in the models, we used various self-reported variables including relationship status, employment status, smoking, and amount of exercise. A participant was considered to use psychotropic medication if she or he had used hypnotics, antidepressants, or tranquilizers during the last year. These questions remained very similar across the different surveys.

Symptoms of insomnia were measured with the question “*During the past 30 days have you had insomnia?* ” with answer options “*often*”, “*sometimes*” *and* “*never.”* Before 2007 symptoms of depression were measured with a question, ““*During the past 30 days how often you have felt depressed?* ” with answer options “*often*”, “*sometimes*” *and* “*never.”* Answer “*often*” was used as an indication of symptoms of depression (5.9% of population). This question was removed after 2002 and in survey of 2007 the question “*Have you had depression diagnosed by a doctor during last year?* ” was used as an indicator of depressive symptoms (7.3% of population).

The questions concerning alcohol use varied between surveys. A participant was considered to be a heavy user of alcohol if he drank spirits several times a week (surveys of 1972–1977), consumed more than 288 g (men) or 192 g (women) of alcohol per week (survey of 1982) or was intoxicated several times a week (surveys of 1987–2012).

### Statistical methods

For survival analyses, Cox proportional hazards regression was used to estimate the risk of suicide conferred by frequent nightmares, and multinomial logistic regression was used to investigate the risk factors for nightmares. Pearson χ^2^ tests were used to test significance of association between categorical variables with Cramer V used to describe the strength of the association. Alpha level of 0.01 was chosen to correct for multiple hypotheses testing in the same data. Analyses were performed with IBM SPSS version 21.

## Results

During the follow-up period there were 20 895 deaths among the 69 101 participants, 398 of which were suicides. Men committed 307 (incidence rate of 43.6/100 000 person-years) and women 91 (11.1/100 000 person-years) of these suicides, and the mean follow-up time from answering the survey to suicide was 13.6 years (SD 9.9, range 0–42.3). Among war veterans, there were 32 suicides (48.8/100 000 person years) during the follow-up period, and the mean time to suicide was 11.2 years (SD 8.0, range 0–27.7).

The prevalence of frequent nightmares in the whole sample, including war veterans, was 3.4% for men and 4.8% for women. Among war veterans the overall prevalence of nightmares was 7.6%. The difference between men and women (Cramer V 0.094, p < 0.001) and between veteran and non-veteran men (Cramer V 0.120, p < 0.001) were statistically significant.

Among veterans who had served at the front and returned home without injury, the prevalence of nightmares was 6.9% (n = 1795), among those who were wounded but made a full recovery it was 7.2% (n = 853) and among those who received permanent disability 10.7% (n = 383). The difference between these veteran groups was statistically significant (Cramer V 0.047, χ^2^(4, n = 3031) = 13.18, p = 0.010). For more detailed analyses about historical trends and prevalence of nightmares among general population and war veterans in these data, see Sandman *et al*.^2^.

Gender, age, relationship status, employment status, smoking, alcohol use, amount of physical exercise, insomnia, symptoms of depression, and use of psychotropic medication were used as covariates in the survival analysis. All of these variables had clear associations with nightmares. The strongest associations were found between nightmares and insomnia (Cramer V 0.251, p < 0.0001), symptoms of depression (Cramer V 0.285, p < 0.0001) and use of psychotropic medication (Cramer V 0.195, p < 0.0001). A full description of these associations can be found in Table S1 in the supplement.

### Nightmares as predictor of suicide

In the Cox regression model, nightmares were a significant predictor of suicide (Table 3). The unadjusted hazard ratio of suicide for persons who reported frequent nightmares compared with those who did not was 2.63 (95% CI [1.80–3.83], p < 0.001). In the fully adjusted model the hazard ratio for frequent nightmares was 1.84 (95% CI [1.15–2.93], p = 0.010) and for occasional nightmares 1.33 (95% CI [1.05–1.69], p = 0.018). When men and women were analysed separately, the mean hazard remained similar, but statistical significance of the results decreased. When identified war veterans or the whole generation who could potentially be war veteran (those born before 1927 and who were at least 18 years old at the end of the war) were excluded from the model, the results did not change significantly. Thus, the results of Tanskanen *et al*.^11^ stand in this extended dataset, and the presence or absence of war veterans does not significantly affect the association between nightmares and suicide. The full analyses can be found in Table S2 and S3 in the supplement.

War veterans could be identified only in the surveys of 1972 and 1977. In a model consisting of male participants of these two surveys and using war experiences as a co-variant, the association between frequent nightmares and suicides was not statistically significant (HR 1.51, 95% CI [0.68–3.36], p = 0.31), probably due to smaller sample size of frequent nightmare sufferers in this subgroup. Occasional nightmares (with larger n) had a weak but statistically significant association with suicides (HR 1.51, 95% CI [1.06–2.15], p = 0.024). These results are presented in Table 2. Full analyses with differently adjusted models can be found in Table S4 in the supplement.

### Risk factors for nightmares among war veterans

We investigated risk factors for frequent nightmares among male war veterans with a multinomial logistic regression model. A model was constructed by starting with 10 predictor variables that were also co-variates in the Cox regression model and by eliminating those with the weakest effect until all predictors in the final model were significant. The model best fitting the data correctly classified 59.5% of the observations (with the chance level being 33.3%), it had a Nagelkerke statistic of 0.175, and was statistically significant (χ^2^(14, n = 3,144) = 351.49, p < 0.0001). The final model included four predictors: symptoms of insomnia (OR for frequent nightmares 14.78 [9.56–22.85]), symptoms of depression (OR 5.70 [3.70–8.79]), heavy use of alcohol (OR 1.50 [0.97–2.31]), and having suffered permanent disability during the war (OR 1.65 [1.06–2.55]). The full model is presented in Table S5 in the supplement.

## Discussion

The original result of Tanskanen *et al*.^11^ did not change in the current extended sample: Nightmares increase the risk for suicide, though the effect size of the association is not large. This is true both for frequent and occasional nightmares and implies a dose dependant risk where increase in nightmare frequency increases the risk for suicide. Nightmares appear to increase the suicide risk among both genders, though in our data women have more nightmares while men have a considerably higher risk for suicide.

Neither of our hypotheses regarding the effect of war veterans were confirmed. Among war veterans, the association between nightmares and suicide risk was not stronger than among the general population. However, the veteran population had higher suicide incidence rate and there was a non-significant trend towards nightmares increasing suicide risk. Therefore, the negative result may partly stem from the lack of power of those analyses confined to the veteran population. However, even if the results had been statistically significant, their magnitude does not appear to differ greatly from that of the general population. Our second hypothesis that excluding war veterans from the whole sample would diminish the association between nightmares and suicide was not supported either.

Together these results suggest that although war veterans experienced more nightmares than the general population, their suicide risk was not significantly higher. However, it must be kept in mind that the veterans in our sample answered the questionnaire over 30 years after the war had ended. Therefore, it is possible that veterans with the most severe PTSD problems and the highest suicide risk had already committed suicide before the FINRISK survey sampling.

Risk factors for frequent nightmares among the war veterans appeared to be similar to the general population in these data^5^, with the exception that a permanent disability acquired during the war was an independent risk factor for having frequent nightmares, even 32–37 years after the presumably traumatic experience of receiving the injury.

Our result support the conclusion of Tanskanen *et al*.^11^ and later studies that nightmares are a risk factor for suicidality and completed suicide. However, as suicides are more common among males, but nightmares among females, and the increased nightmare prevalence among war veterans does not translate to increased suicide rate, our results point towards a conclusion that the predictive power of nightmares is quite limited. There must be other stronger risk factors for suicide that may override the risk factors measured by questions about nightmares. However, as nightmares are potentially modifiable risk factor for suicide, they should not be overlooked in suicide prevention.

Our study is subject to some limitations. First, war veterans could only be identified in a subset of the whole sample, and this diminishes the statistical power of the analyses conducted on veterans only. Among participants who entered the study in surveys conducted later than 1977, there are also unidentified war veterans who were born before 1927. Although they could be excluded from analyses by excluding participants by their birth year, they could not be identified and used in analyses that specifically targeted veterans. We also do not have information about the actual traumatic experiences of this population and can only presume that the incidence of traumatic experiences among veterans is higher than among general population, similarly to their nightmare frequency.

Another limitation of this kind of a study design is the length of the follow-up period. For most of the people who committed suicide during the follow-up, several years had passed since participating in the FINRISK survey. If we assume that frequent nightmares reported several years prior to suicide are a risk factor for suicide, we must also assume that either nightmares are a stable trait over time or they are a manifestation of some underlying risk factor, like vulnerability to depression. Some evidence exists that nightmares might be a stable trait from childhood to adulthood^6^, but more research on the topic is warranted.

The study would also have benefitted from more exact measures of nightmares, insomnia and depression and especially from questions addressing PTSD symptoms. The participants were not provided with a definition of nightmare and as a result, we cannot differentiate between nightmares that cause an awakening and bad dreams that do not.

Lastly, during the 40 years the FINRISK data has been collected, wording of some of the questions addressing depression and alcohol use have changed between surveys. Though this poses a problem for the validity of the co-variates in question, we decided that it is more important to use some measure of these known correlates of nightmares and suicide as a co-variate in the model than to aim for the highest validity.

The main strength of the study lies in the unique population level sample spanning a long time period and including war veterans. Although the re-examination of the data with identified war veterans did not change the results of the original study, we improved the original design by examining the veteran population separately and validated the association between nightmares and suicide in Finland. We feel that these types of research efforts aimed at improving reliability are very important, especially now that the replicability of results in psychological science has been questioned^27^.

Our study contributes to the converging evidence that nightmares are a predictor of suicide. Although the question of whether nightmares have a primary effect, or whether they are an indicator of some other underlying risk factor, remains unanswered, nightmares should be considered an early warning sign in suicide prevention.

## Additional Information

**How to cite this article:** Sandman, N. *et al*. Nightmares as predictors of suicide: an extension study including war veterans. *Sci. Rep.* **7**, 44756; doi: 10.1038/srep44756 (2017).

**Publisher's note:** Springer Nature remains neutral with regard to jurisdictional claims in published maps and institutional affiliations.

## Supplementary Material

Supplementary Information

## Figures and Tables

**Table 1:** Studies investigating the association between nightmares and suicide.

Study	Participants	Measure of suicidality	Measure of nightmares	Effect of nightmares on suicidality
Ağargün *et al*.^28^	63 depressed patients	SADS suicide subscale	Interview	Nightmares related to high suicidality
Ağargün *et al*.^29^	100 depressed patients	Suicide attempt	Interview	Nightmares common among suicide attempters
Bernert *et al*.^30^	176 psychiatric outpatients	BSS suicidal ideation scale	DDNSI questionnaire	Nightmares related to high suicidality
Cukrowicz *et al*.^31^	222 students	DSISS suicidal ideation subscale	DDNSI questionnaire	Nightmares related to high suicidality
Liu 2004^32^	1,362 adolescents	Self-assessment	Self-assessment	OR 1.69 for suicide ideation
Li *et al*. 2010^33^	1,231 psychiatric outpatients	Suicide attempt	Self-assessment	OR 8.17 for suicide attempt
Li *et al*.^34^	388 schizophrenic outpatients	Suicide attempt	Self-assessment	Nightmares & insomnia, HR 7.43 for suicide attempt
Nadorff *et al*.^35^,^36^	583–673 students	SBQ questionnaire	DDNSI questionnaire	Nightmares related to high suicidality
Nadorff *et al*.^37^	81 adults over the age of 65	GSIS questionnaire	DDNSI questionnaire	No significant effect
Sjöström *et al*.^38^,^39^	165 suicidal patients	Suicide assessment scale	Self-assessment	OR 2.55 for high suicidality, OR 3.15 for suicide attempt
Tanskanen *et al*.^11^	36,211 general adult population	Completed suicide	Self-assessment	HR 2.05 for suicide
Wong *et al*.^40^	392 adolescents from alcoholic families	YSR questionnaire suicidal ideation items	Self-assessment	No significant effect

**Table 2:** Number of participants and suicides in different sub-groups of the data.

Group	N	Suicides	Incidence rate
Whole sample	71068	398	26.2
Men	34059 (48.4%)	307	43.6
Women	36611 (51.6%)	91	11.1
Participants born before 1927	11214	63	24.1
Men	5223 (46.6%)	49	46.8
Women	5991 (53.4%)	14	8.9
War veterans	3307	32	48.8
Men	3144 (95.1%)	31	48.8
Women	163 (4.9%)	1	n/a

Whole sample includes both subgroups and those born before 1927 includes war veteran group. Incidence rate is suicide/100 000 person years.

**Table 3:** Cox regression model of suicide risk according to frequency of nightmares.

Nightmares	Hazard Ratio	95% CI	P	N of suicides	N
**RISK OF SUICIDE: WHOLE SAMPLE**^**1**^					
Often	1.84	1.15–2.93	0.010	26	2174
Occasionally	1.33	1.05–1.69	0.018	149	21774
Not at all	1			157	31252
**Total**				332	54815
**RISK OF SUICIDE: MEN OF 1972 AND 1977**^**2**^					
Often	1.51	0.68–3.36	0.308	8	401
Occasionally	1.51	1.06–2.15	0.024	64	3253
Not at all	1			72	6169
**War experiences**					
Served at front	1.37	0.77–2.45	0.282	25	2695
Did not serve at front	1			119	7264
**Total**				144	9815

^1^Adjusted with sex, age, relationship status, employment status, smoking, use of alcohol, amount of exercise, symptoms of insomnia, symptoms of depression and use of psychotropic medication.

^2^Adjusted with age, relationship status, employment status, smoking, use of alcohol, amount of exercise, symptoms of insomnia, symptoms of depression and use of psychotropic medication.
