# Supplementary material for: Nightmares as predictors of suicide: an extension study including war veterans
Source: Sci Rep. 2017 Mar 15;7:44756. doi: 10.1038/srep44756 (PMC5353666; doi:10.1038/srep44756)
Supplement: Supplementary Information [file srep44756-s1.pdf]

Nightmares as predictors of suicide: an extension study including war veterans

Nils Sandman, Katja Valli, Erkki Kronholm, Erkki Vartiainen, Tiina Laatikainen & Tiina Paunio

Supplemental material

Contents

TABLE S1. Association between nightmares and covariates 2
TABLE S2. Nightmares as predictors for suicide 3
TABLE S3. Nightmares as predictors for suicide, Model 3 5
TABLE S4. Nightmares as predictors of suicide among war veterans 6
TABLE S5. Risk factors of nightmares among war veterans 7

TABLE S1. Association between nightmares and covariates

	Nightmares Often	Sometimes	Never	N	Cramer V	χ^2
**Gender**				69 101	0.094	<0.0001
Women	4.8%	43.8%	51.4%	35 621 (51.6%)		
Men	3.4%	35.9%	60.7%	33 480 (48.4%)		
**In relationship**				63 585	0.027	<0.0001
Yes	3.9%	39.3%	56.8%	47 828 (75%)		
No	4.9%	40.6%	54.5%	15 757 (25%)		
**Employed**				67 284	0.115	<0.0001
Yes	3.0%	38.1%	59.0%	46 974 (30.6%)		
No	6.7%	44.4%	48.9%	20 310 (69.4%)		
**Smoking**				63 072	0.032	<0.0001
Yes	4.9%	41.0%	54.1%	17 239 (27.1%)		
No	3.8%	39.1%	57.1%	45 833 (72.9%)		
**Alcohol use**				63 409	0.045	<0.0001
Several times a week	9.3%	47.1%	43.6%	1376 (2.2%)		
Occasionally	3.7%	41.1%	55.2%	42 549 (66.7%)		
Not at all	4.4%	36.9%	58.7%	19 484 (31.1%)		
**Physical exercise**				66 996	0.037	<0.0001
Weekly or more often	3.5%	40.4%	56.1%	44 424 (66.2%)		
Seldom	5.1%	39.2%	55.8%	22 572 (33.8%)		
**Insomnia**				68 607	0.251	<0.0001
Often	19.1%	4.9%	1.5%	5312 (7.9%)		
Sometimes	53.3%	53.5%	30.1%	23 396 (34.3%)		
Never	27.6%	41.6%	68.4%	39 899 (57.8%)		
**Symptoms of depression**				63 150	0.285	<0.0001
Yes	25.2%	52.9%	21.9%	3588 (5.8%)		
No	2.8%	38.6%	58.5%	59 562 (94.2%)		
**Psychotropic medication**				69 101	0.195	<0.0001
Yes	12.3%	52.1%	35.6%	8453 (12.2%)		
No	3.0%	38.3%	58.7%	60 648 (87.8%)		

TABLE S2. Nightmares as predictors for suicide

Cox regression model.

	Hazard Ratio	95% CI	P	Suicides	N
**Model 1**				382	68 646
NM often	2.63	1.80 – 3.83	<0.001		
NM sometimes	1.49	1.21 – 1.83	<0.001		
NM never	1				
**Model 2**				382	68 646
NM often	3.50	2.93 – 5.12	<0.001		
NM sometimes	1.71	1.39 – 2.11	<0.001		
NM never	1				
**Model 3**				332	54 815
NM often	1.84	1.15 – 2.93	0.010		
NM sometimes	1.33	1.05 – 1.69	0.018		
NM never	1				
**Model 4 women**				78	27 726
NM often	1.93	0.90 – 4.12	0.091		
NM sometimes	0.89	0.53 – 1.47	0.64		
NM never	1				
**Model 4 men**				254	27 064
NM often	1.56	0.86 – 2.89	0.143		
NM sometimes	1.48	1.13 – 1.96	0.004		
NM never	1				
**Model 5**				306	67 761
NM often	2.00	1.22 – 3.28	0.006		
NM sometimes	1.37	1.07 – 1.75	0.013		
NM never	1				
**Model 5 men**				229	31 222
NM often	1.72	0.87 – 3.38	0.118		
NM sometimes	1.54	1.16 – 2.04	0.003		
NM never	1				
**Model 6**				281	59 854
NM often	2.01	1.19 – 3.41	0.009		
NM sometimes	1.38	1.07 – 1.79	0.013		
NM never	1				

NM = Nightmares

Reference category for nightmares is “no nightmares during the last 30 days”

Model 1: Unadjusted.

Model 2: Adjusted with sex and age.

Model 3: Adjusted with sex, age, relationship status, employment status, smoking, use of alcohol, amount of exercise, symptoms of insomnia, symptoms of depression and use of psychotropic medication.

Model 4: Men and women separately. Adjusted with age, relationship status, employment status, smoking, use of alcohol, amount of exercise, symptoms of insomnia, symptoms of depression and use of psychotropic medication.

Model 5: Known war veterans excluded. Adjusted with sex, age, relationship status, employment status, smoking, use of alcohol, amount of exercise, symptoms of insomnia, symptoms of depression and use of psychotropic medication.

Model 5 men: Men only, known war veterans excluded. Adjusted with age, relationship status, employment status, smoking, use of alcohol, amount of exercise, symptoms of insomnia, symptoms of depression and use of psychotropic medication.

Model 6: All participants born before 1927 excluded – data does not include any war veterans. Adjusted with sex, age, relationship status, employment status, smoking, use of alcohol, amount of exercise, symptoms of insomnia, symptoms of depression and use of psychotropic medication.

TABLE S3. Nightmares as predictors for suicide, Model 3.

Cox regression model with all co-variants displayed.

RISK OF SUICIDE: WHOLE SAMPLE, N = 54815				
	**Hazard Ratio**	**95% CI**	**P**	**N**
**Nightmares**				
Often	1.84	1.15 – 2.93	0.010	2174
Occasionally	1.33	1.05 – 1.69	0.018	21774
Not at all	1			31252
**Insomnia**				
Often	1.42	0.94 – 2.16	0.097	4014
Occasionally	1.30	1.01 – 1.67	0.041	18188
Not at all	1			32613
**Gender**				
Male	3.52	2.68 – 4.61	<0.001	27089
Female	1			27726
**Relationship status**				
Not in relationship	1.31	1.03 – 1.66	0.027	13183
In relationship	1			41632
**Employment**				
Unemployed	1.36	1.03 – 1.79	0.030	14994
Employed	1			39821
**Smoking**				
Smokes	1.96	1.57 – 2.50	<0.001	15677
Does not smoke	1			39138
**Alcohol use**				
Heavy	1.24	0.73 – 2.13	0.426	1196
Moderate	0.98	0.74 – 1.31	0.907	36992
None	1			16627
**Exercise**				
Regular	1.23	0.99 – 1.54	0.61	35839
Seldom				18976
**Depression**				
Symptoms of depression	0.95	0.62 – 1.44	0.801	3002
No symptoms of depression	1			51813
**Psychotropic medication**				
Yes	1.91	1.38 – 2.63	<0.001	6482
No	1			48333

TABLE S4. Nightmares as predictors of suicide among war veterans

Cox regression model, men of surveys in 1972 and 1977 only.

	Hazard Ratio	95% CI	P	N of suicides	Total N
**Model 1**				165	10 908
NM Often	1.96	0.94 – 4.07	0.072		
NM Occasionally	1.79	1.31 – 2.45	<0.001		
War veteran	1.25	0.83 – 1.86	0.285		
**Model 2**				165	10 908
NM Often	1.96	0.94 – 4.08	0.071		
NM Occasionally	1.79	1.31 – 2.45	<0.001		
War veteran	1.22	0.71 – 2.09	0.480		
**Model 3**				144	9815
NM Often	1.51	0.68 – 3.36	0.308		
NM Occasionally	1.51	1.06 – 2.15	0.024		
War veteran	1.37	0.77 – 2.45	0.282		

NM = Nightmares

Reference category for nightmares is “no nightmares during the last 30 days” and for war veterans “no service at the front during WWII”

Model 1: Adjusted with war experiences.

Model 2: Adjusted with war experiences and age.

Model 3: Adjusted with , war experiences, age, relationship status, employment status, smoking, use of alcohol, amount of exercise, symptoms of insomnia, symptoms of depression and use of psychotropic medication.

TABLE S5. Risk factors of nightmares among war veterans

Multinominal logistic regression model of risk factors for nightmares among war veterans.

Variable		Frequent nightmares			Occasional nightmares		
		OR	OR 95% CI	p	OR	OR 95% CI	p
**Symptoms of insomnia**	Often	14.78	9.56 – 22.85	<0.001	3.38	2.51 – 4.54	<0.001
	Sometimes	3.31	2.25 – 4.86	<0.001	2.49	2.01 – 2.95	<0.001
	Never	1			1		
**Symptoms of depression**	Yes	5.70	3.70 – 8.79	<0.001	2.28	1.61 – 3.22	<0.001
	No	1			1		
**Spirits several times a week**	Yes	2.56	1.29 – 5.21	0.008	1.96	1.26 – 3.04	0.003
	Spirits less often	1.50	0.97 – 2.31	0.067	1.60	1.28 – 1.99	<0.001
	Does not use alcohol	1			1		
**Wounded during the war**	Yes, war invalid	1.65	1.06 – 2.55	0.026	1.31	1.02 – 1.68	0.032
	Yes, full recovery	0.97	0.68 – 1.39	0.872	1.14	0.95 – 1.36	0.163
	No	1			1		

Reference category for nightmares is “no nightmares during the last 30 days”
